# Association between triglyceride-glucose index and sarcopenia in older adults without either dysglycemia or central obesity: a cross-sectional study from CHARLS

**DOI:** 10.1515/med-2026-1487

**Published:** 2026-07-07

**Authors:** Jiaojiao Lin, Zhaozhao Zhu

**Affiliations:** Department of Endocrinology, Quanzhou First Hospital Affiliated to Fujian Medical University, Quanzhou, Fujian, China

**Keywords:** sarcopenia, triglyceride-glucose index, older adults, lipid metabolism, healthy aging

## Abstract

**Objectives:**

This study investigated the association between the triglyceride-glucose (TyG) index and sarcopenia in a metabolically healthier older population.

**Methods:**

This cross-sectional study utilized data from China Health and Retirement Longitudinal Study (CHARLS), involving 1,544 participants aged ≥60 years who were free of both dysglycemia (i.e., diabetes or prediabetes) and central obesity. Logistic regression was used to evaluate the association between the TyG index and sarcopenia.

**Results:**

Participants with sarcopenia had a significantly lower TyG index than those without sarcopenia (8.29 [8.01–8.59] vs. 8.38 [8.07–8.69]; p=0.001), and the prevalence of sarcopenia declined progressively across TyG quartiles (Q1–Q4: 54.3 %, 53.4 %, 47.4 %, 43.8 %; p for trend=0.001). After adjustment for significant covariates, a higher TyG index was independently associated with lower odds of sarcopenia (per quartile: OR = 0.826, 95 % CI: 0.720–0.947; per unit: OR = 0.714, 95 % CI: 0.517–0.985). Compared with the lowest quartile, participants in the highest quartile had a 44.8 % lower odds of sarcopenia (OR = 0.552, 95 % CI: 0.357–0.852).

**Conclusion:**

Among older adults without either dysglycemia or central obesity, a higher TyG index was independently associated with a lower odds of sarcopenia.

## Introduction

Sarcopenia is a geriatric syndrome characterized by progressive loss of muscle mass, strength, and function with advancing age. The prevalence of sarcopenia among older adults in Asian countries ranges from 6.8 to 25.7 %, with higher rates observed in older age groups [1], 2]. Accumulating evidence demonstrates that sarcopenia is associated with cardiovascular disease and increases the risk of adverse events such as frailty, falls, fractures, disability, and mortality [3], 4].

The triglyceride-glucose (TyG) index is calculated from fasting triglyceride and glucose levels and was originally used as a surrogate marker of insulin resistance [5], [6], [7]. Insulin resistance represents a core mechanism underlying metabolic disorders and may increase the risk of sarcopenia by influencing skeletal muscle synthesis and degradation through multiple pathways [8]. Previous studies have reported a positive association between the TyG index and sarcopenia, particularly in individuals with obesity or diabetes – conditions commonly accompanied by insulin resistance – in whom this association appears to be stronger [9], 10]. However, in studies involving the general middle-aged and older population, the relationship between the TyG index and sarcopenia has not always been consistent. Some studies have reported a positive association between the two [11], [12], [13], [14], [15], whereas others have found an inverse association [16], [17], [18], [19]. This inconsistency may be partly explained by the fact that the triglyceride component of the TyG index not only reflects insulin resistance, but also, as the body’s principal form of energy storage, may indicate overall energy and nutritional status [20], [21], [22], [23].

Based on this, the present study focused on older adults without either dysglycemia (i.e., diabetes or prediabetes) or central obesity to examine the independent association between the TyG index and sarcopenia. This metabolically healthier subgroup was selected to minimize confounding from insulin resistance and central obesity, thereby clarifying the intrinsic relationship between the TyG index and sarcopenia.

## Materials and methods

### Study design and participants

**Figure 1:**
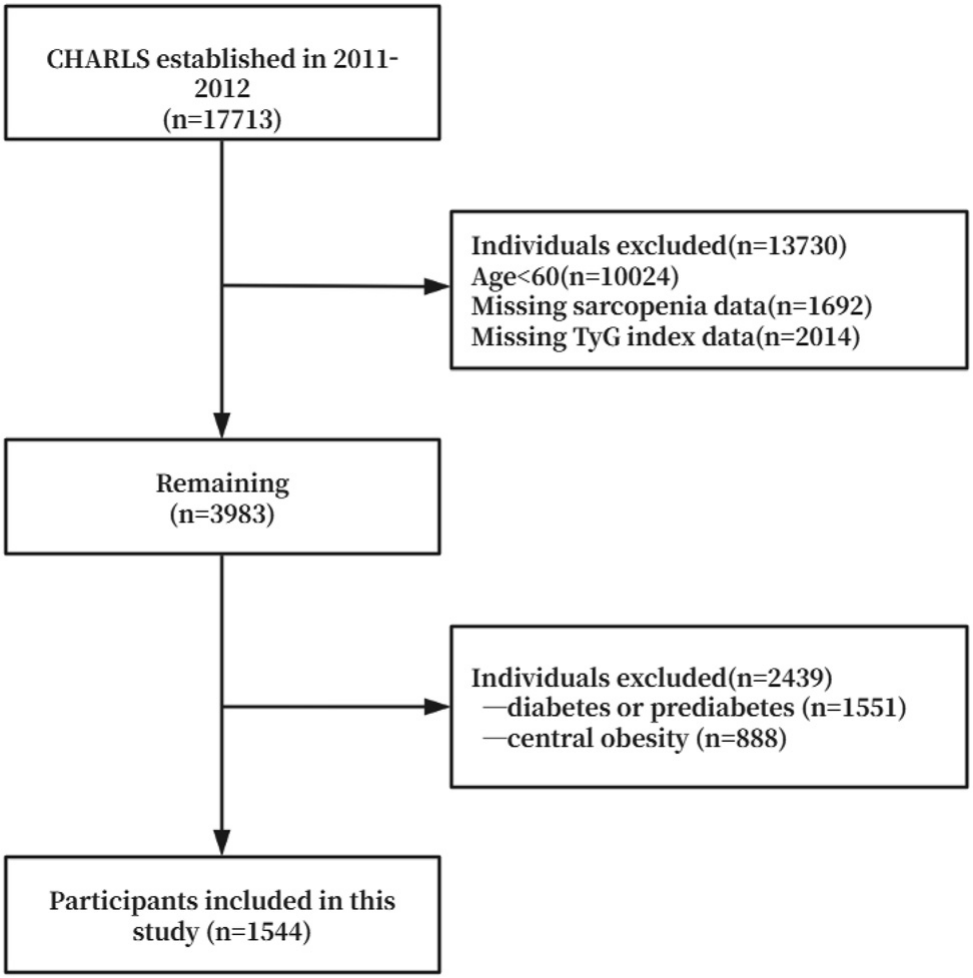
Flow diagram of participant enrollment. Abbreviations: CHARLS, China health and retirement longitudinal study.

The China Health and Retirement Longitudinal Study (CHARLS) is a longitudinal, nationally representative survey designed to investigate the aging process in the Chinese population aged 45 and above. The baseline survey was conducted during 2011–2012, recruiting over 17,000 participants from 450 communities or villages across 150 counties/districts in 28 provinces. Detailed descriptions of the sampling procedures, questionnaire design, and clinical assessment methods have been reported elsewhere [24]. The study was conducted in accordance with the Declaration of Helsinki (as revised in 2013), and approved by the Institutional Review Board of of Peking University (IRB00001052-11015). Informed consent was obtained from all individuals included in this study, or their legal guardians or wards. The present cross-sectional analysis drew on 17,713 participants from the CHARLS 2011–2012 baseline survey. We excluded (1) individuals aged <60 years (n=10,024); (2) those missing sarcopenia data (n=1,692); and (3) those missing TyG index data (n=2,014). After applying criteria (1)–(3), 3,983 participants remained. We further excluded (4) individuals with dysglycemia (i.e., diabetes or prediabetes; n=1,551) and (5) those with central obesity (n=888), yielding a final analytic sample of 1,544 adults aged 60 years and older without dysglycemia, without central obesity, and without missing key variables, representing only 8.7 % of the original sample. The detailed participant selection process is shown in Figure 1.

### Sarcopenia

According to the 2019 consensus of the Asian Working Group for Sarcopenia (AWGS), the assessment of sarcopenia involves three components: appendicular skeletal muscle mass (ASM), muscle strength, and physical performance [1]. Sarcopenia is defined as low ASM accompanied by reduced muscle strength or declined physical performance. In this study, ASM was estimated using a previously validated equation in a Chinese population [25]:

$$\text{ASM}=0.193\times \text{weight }\left(\text{kg}\right)+0.107\times \text{height }\left(\text{cm}\right)-4.157\times \text{sex }\left(\text{male}=1,\text{female}=2\right)-0.037\times \text{age }\left(\text{years}\right)-2.631$$

This formula incorporates age, sex, height, and weight, and the ASM was adjusted for height squared to obtain the ASM index (ASM/Ht^2^). Low muscle mass was defined as ASM/Ht^2^ below the sex-specific 20th percentile. Previous studies have demonstrated a high level of agreement between this method and dual-energy X-ray absorptiometry (DXA) [25]. The cutoff values used in this study were ASM/Ht^2^ <5.27 kg/m^2^ for women and <7.00 kg/m^2^ for men. Handgrip strength was measured using a “Yuejian™ WL-1000” dynamometer, and the maximum value was used for analysis. Reduced muscle strength was defined as handgrip strength <28 kg for men and <18 kg for women. Physical performance was evaluated by gait speed, the five-times chair stand test, and balance tests in three stances (side-by-side, semi-tandem, and tandem). The Short Physical Performance Battery (SPPB), consisting of gait speed, chair stand time, and balance tests, was used to quantify physical performance, with each component scored up to 4 points for a total of 12 points. Impaired physical performance was defined as gait speed <1.0 m/s, chair stand time ≥12 s, or SPPB <9.

### TyG index

Venous blood samples were collected from participants by medical personnel from the Chinese Center for Disease Control and Prevention (China CDC). Fasting status was determined and recorded for each participant. After centrifugation, the venous blood samples were transported to the laboratory of Capital Medical University for further analysis. Fasting serum triglyceride and plasma glucose levels were measured using an enzymatic colorimetric method. The TyG index was subsequently calculated using the following formula [26]:

$$\text{Ty}G=\mathrm{Ln}\;\left[\;\left(\text{triglyceride }\left(\text{mg }/\text{dL}\right)\times \text{glucose }\left(\text{mg }/\text{ dL}\right)\;/\;2\;\right)\;\right]$$

### Dysglycemia and central obesity

Dysglycemia, including diabetes and prediabetes, was defined as meeting any of the following criteria [27], 28]: (1) fasting plasma glucose ≥110 mg/dL; (2) glycated hemoglobin ≥5.7 %; or (3) self-reported physician diagnosis of diabetes or elevated blood glucose. The fasting plasma glucose threshold was based on the 1999 World Health Organization (WHO) criteria and was aligned with the CHARLS definition of self-reported elevated blood glucose. According to the criteria recommended by the Working Group on Obesity in China (WGOC) [29], central obesity was defined as a waist circumference of ≥90 cm in men and ≥85 cm in women.

### Covariates

Trained interviewers collected information on participants’ sociodemographic characteristics and health behaviors, including age, sex, educational level, marital status, place of residence, engagement in daily physical activities, smoking status, and alcohol consumption, using a structured questionnaire. Each participant then underwent a physical examination and venous blood sampling. We obtained anthropometric and clinical measurements, including height, weight, waist circumference, systolic blood pressure (SBP), diastolic blood pressure (DBP), white blood cell count, hemoglobin, serum creatinine, blood urea nitrogen, C-reactive protein, blood glucose, glycated hemoglobin, triglyceride, total cholesterol, low-density lipoprotein cholesterol (LDL-C), and high-density lipoprotein cholesterol (HDL-C). Because the TyG index is calculated using fasting plasma glucose and fasting triglyceride levels, only participants in the fasting state were included in the present study; non-fasting participants were automatically excluded because these two measurements were unavailable. Body mass index (BMI, kg/m^2^) was calculated as weight (kg) divided by height squared (m^2^).

### Statistical analysis

Statistical analyses were performed using SPSS software, version 26.0 (IBM Corp., Armonk, NY, USA). The distribution of continuous variables was assessed using the Kolmogorov–Smirnov test. Variables with a normal distribution are presented as the mean ± standard deviation and were compared between groups using the independent samples t-test. Variables with a non-normal distribution are presented as the median [interquartile range] and were compared using the Mann–Whitney *U* test. Categorical variables are expressed as numbers (percentages) and were compared between groups using the *χ* ^2^ test. Logistic regression was used to evaluate the association between the TyG index and sarcopenia. p<0.05 was considered statistically significant.

**Research ethics:** The study was conducted in accordance with the Declaration of Helsinki (as revised in 2013), and approved by the Institutional Review Board of of Peking University (IRB00001052-11015).

**Informed consent:** Informed consent was obtained from all individuals included in this study, or their legal guardians or wards.

## Results

### Baseline characteristics of the study participants

Table 1 presents the characteristics of the study participants according to sarcopenia status. Among the 1,544 participants, the prevalence of sarcopenia was 49.7 % (768/1,544). Compared with those without sarcopenia, participants with sarcopenia were older (median [IQR], 70 [65–75] vs. 64 [62–69] years; p<0.001), more often female (49.2 vs. 30.5 %; p<0.001), had a lower educational level (primary school or below: 89.7 vs. 82.1 %; p<0.001), were more likely to be unmarried (23.2 vs. 14.7 %; p<0.001), and were more likely to live in rural areas (90.5 vs. 86.4 %; p=0.038). In addition, participants with sarcopenia had a significantly lower TyG index (8.29 [8.01–8.59] vs. 8.38 [8.07–8.69]; p=0.001), along with lower BMI (19.2 [18.0–20.1] vs. 22.2 [21.1–23.5] kg/m^2^; p<0.001), waist circumference (75.1 [71.0–79.0] vs. 81.0 [78.0–84.0] cm; p<0.001), triglyceride (83.2 [63.7–110.6] vs. 91.2 [66.6–123.0] mg/dL; p=0.002), and LDL-C levels (109.8 [88.1–132.6] vs. 114.4 [94.3–133.4] mg/dL; p=0.015), and higher HDL-C levels (57.2 [47.9–68.8] vs. 53.0 [44.1–62.6] mg/dL; p<0.001).

**Table 1:** Comparison of baseline characteristics between the non-sarcopenia and sarcopenia groups.

Characteristics	Total, n=1,544	Non-sarcopenia group, n=776	Sarcopenia group, n=768	p-Value
Age, years	67 [63–73]	64 [62–69]	70 [65–75]	<0.001
Sex, n (%)				<0.001
Male	929 (60.2)	539 (69.5)	390 (50.8)	
Female	615 (39.8)	237 (30.5)	378 (49.2)	
Educational level, n (%)				<0.001
Primary education or below	1,326 (85.9)	637 (82.1)	689 (89.7)	
Above primary education	218 (14.1)	139 (17.9)	79 (10.3)	
Marital status, n (%)				<0.001
Unmarried	1,252 (81.1)	662 (85.3)	590 (76.8)	
Married	292 (18.9)	114 (14.7)	178 (23.2)	
Residence, n (%)				0.039
Rural	896 (88.4)	456 (86.4)	440 (90.5)	
Urban	118 (11.6)	72 (13.6)	46 (9.5)	
Physical activity, n (%)				0.179
Yes	540 (35.0)	284 (36.6)	256 (33.3)	
No	1,004 (65.0)	492 (63.4)	512 (66.7)	
Smoking, n (%)				<0.001
Yes	763 (49.4)	423 (54.5)	340 (44.3)	
No	781 (50.6)	353 (45.5)	428 (55.7)	
Drinking, n (%)				0.003
Yes	540 (35.0)	299 (38.5)	241 (31.4)	
No	1,004 (65.0)	477 (61.5)	527 (68.6)	
BMI, kg/m2	20.6 [19.1–22.3]	22.2 [21.1–23.5]	19.2 [18.0–20.1]	<0.001
Waist circumference, cm	78.0 [73.4–82.1]	81.0 [78.0–84.0]	75.1 [71.0–79.0]	<0.001
SBP, mmHg	126.0 [113.0–142.5]	126.0 [113.5–141.5]	126.0 [112.5–142.5]	0.563
DBP, mmHg	72.0 [64.0–79.5]	72.5 [65.5–80.5]	71.0 [63.0–78.5]	<0.001
White blood cells, 109/L	5.8 [4.8–7.0]	5.8 [4.8–7.0]	5.8 [4.7–6.9]	0.513
Hemoglobin, g/dL	13.9 [12.7–15.1]	14.3 [13.1–15.5]	13.5 [12.4–14.7]	<0.001
Serum creatinine, mg/dL	0.79 [0.68–0.92]	0.81 [0.70–0.94]	0.76 [0.65–0.89]	<0.001
Blood urea nitrogen, mg/dL	15.9 [13.1–19.1]	15.8 [13.1–19.1]	16.0 [13.1–19.1]	0.627
C-reactive protein, mg/L	0.96 [0.53–2.07]	0.99 [0.55–1.92]	0.92 [0.52–2.20]	0.715
Fasting plasma glucose, mg/dL	97.2 [91.3–102.6]	97.7 [92.3–102.4]	96.7 [90.7–102.8]	0.134
Glycated hemoglobin, %	5.1 [4.8–5.3]	5.1 [4.9–5.3]	5.1 [4.8–5.3]	0.171
Triglyceride, mg/dL	86.7 [65.5–115.1]	91.2 [66.6–123.0]	83.2 [63.7–110.6]	0.002
Total cholesterol, mg/dL	187.1 [164.0–210.6]	186.0 [165.1–209.8]	188.1 [162.4–211.6]	0.903
LDL-C, mg/dL	112.1 [91.6–133.0]	114.4 [94.3–133.4]	109.8 [88.1–132.6]	0.015
HDL-C, mg/dL	55.3 [46.0–65.3]	53.0 [44.1–62.6]	57.2 [47.9–68.8]	<0.001
TyG	8.34 [8.04–8.64]	8.38 [8.07–8.69]	8.29 [8.01–8.59]	0.001

Values are presented as median [IQR] or number (%). Continuous variables were compared using Mann-Whitney *U* test; categorical variables were compared using *χ* ^2^ test. p<0.05 was considered statistically significant. BMI, body mass index; SBP: systolic blood pressure; DBP: diastolic blood pressure; LDL-C, low-density lipoprotein cholesterol; HDL-C, high-density lipoprotein cholesterol; TyG, triglyceride-glucose index. Prevalence of sarcopenia across TyG index quartiles.

The TyG index was categorized into quartiles (Q1–Q4), as shown in Table 2. The TyG index in Q1 was 7.86 [7.72–7.96], with a sarcopenia prevalence of 54.3 %; in Q2 it was 8.18 [8.11–8.25], with a prevalence of 53.4 %; in Q3 it was 8.48 [8.40–8.55], with a prevalence of 47.4 %; and in Q4 it was 8.88 [8.75–9.09], with a prevalence of 43.8 %. The prevalence of sarcopenia significantly decreased across increasing TyG index quartiles (Q1 54.3 % vs. Q2 53.4 % vs. Q3 47.4 % vs. Q4 43.8 %; Cochran–Armitage trend test: *χ* ^2^ = 11.038, p for trend=0.001).

**Table 2:** Prevalence of sarcopenia across TyG index quartiles.

	Total (n=1,544)	Q1 (n=386)	Q2 (n=386)	Q3 (n=386)	Q4 (n=386)	p-Value
TyG	8.34 [8.04–8.64]	7.86 [7.72–7.96]	8.18 [8.11–8.25]	8.48 [8.40–8.55]	8.88 [8.75–9.09]	–
Sarcopenia, n (%)	768 (49.7)	210 (54.3)	206 (53.4)	183 (47.4)	169 (43.8)	0.001

Values are presented as median [IQR] or number (%). The TyG index was categorized into quartiles (Q1–Q4). The prevalence of sarcopenia across TyG index quartiles was compared using the Cochran–Armitage trend test. P <0.05 was considered statistically significant. TyG, triglyceride-glucose index.

### Association between TyG index and sarcopenia based on logistic regression analysis

Sarcopenia status (yes = 1, no = 0) was used as the dependent variable, and the TyG index (continuous) and its quartiles (entered both as ordinal and categorical variables) were used as independent variables in logistic regression models, as shown in Table 3. A higher TyG index was associated with lower odds of sarcopenia. Specifically, for each one-quartile increase in the TyG index, the odds of sarcopenia were 14.1 % lower (OR=0.859, 95 % CI: 0.785–0.940, p=0.001), and for each one-unit increase in the TyG index, the odds were 27.2 % lower (OR=0.728, 95 % CI: 0.586–0.905, p=0.004). Compared with participants in the lowest TyG quartile, those in the highest quartile had 34.7 % lower odds of sarcopenia (OR=0.653, 95 % CI: 0.491–0.867, p=0.003). In subsequent multivariable logistic regression models, covariates were selected based on variables showing statistically significant differences between participants with and without sarcopenia. Variables considered included age, sex, educational level, marital status, residence, smoking, drinking, DBP, hemoglobin, serum creatinine, BMI, and waist circumference. Height, weight, triglyceride, LDL-C, and HDL-C were excluded from the final model to avoid collinearity. After adjustment for these covariates, the TyG index remained independently associated with a lower odds of sarcopenia. For each one-quartile increase in the TyG index, the odds of sarcopenia decreased by 17.4 % (OR=0.826, 95 % CI: 0.720–0.947, p=0.006), and for each one-unit increase, by 28.6 % (OR=0.714, 95 % CI: 0.517–0.985, p=0.040). Compared with the lowest quartile, participants in the third quartile had 36.2 % lower odds of sarcopenia (OR = 0.638, 95 % CI: 0.422–0.964, p=0.033), and those in the highest quartile had 44.8 % lower odds (OR = 0.552, 95 % CI: 0.357–0.852, p=0.007).

**Table 3:** Association between TyG index and sarcopenia based on logistic regression analysis.

TyG	Model 1 OR (95 % CI)	Model 2 OR (95 % CI)	Model 3 OR (95 % CI)	Model 4 OR (95 % CI)
Per-quartile increase	0.859 (0.785–0.940)^b^	0.763 (0.673–0.866)^c^	0.795 (0.697–0.906)^b^	0.826 (0.720–0.947)^b^
Per-unit increase	0.728 (0.586–0.905)^b^	0.568 (0.419–0.769)^c^	0.639 (0.468–0.873)^b^	0.714 (0.517–0.985)^a^
Q1	Reference	Reference	Reference	Reference
Q2	0.959 (0.723–1.273)	0.670 (0.457–0.982)^a^	0.693 (0.468–1.027)	0.717 (0.477–1.077)
Q3	0.756 (0.569–1.003)	0.565 (0.385–0.829)^b^	0.624 (0.420–0.925)^a^	0.638 (0.422–0.964)^a^
Q4	0.653 (0.491–0.867)^b^	0.431 (0.289–0.643)^c^	0.481 (0.317–0.730)^b^	0.552 (0.357–0.852)^b^

Results are presented as odds ratios (ORs) with 95 % confidence intervals (CIs). The TyG index was categorized into quartiles (Q1–Q4). Categorical variables were converted into dummy variables as appropriate for inclusion in the regression models. Associations between independent variables and the outcome were evaluated using logistic regression models. pP0.05 was considered statistically significant. Model 1: Unadjusted model. Model 2: Adjusted for age, sex, educational level, marital status, residence. Model 3: further adjusted for smoking, drinking, DBP, hemoglobin, and creatinine based on model 2. Model 4: further adjusted for BMI, and waist circumference based on model 3. ^a^p<0.05, ^b^p<0.01, ^c^p<0.001. TyG, triglyceride-glucose index; OR, odds ratio; CI, confidence interval; DBP, diastolic blood pressure; BMI, body mass index.

## Discussion

In this cross-sectional study based on CHARLS data, we for the first time systematically examined the association between the TyG index and sarcopenia among Chinese adults aged 60 years and older who were free of both dysglycemia and central obesity. In our results, participants with sarcopenia had a lower TyG index than those without, and the prevalence of sarcopenia decreased markedly from the lowest to the highest TyG quartile (Q1 to Q4), indicating an inverse association between higher TyG levels and sarcopenia. Consistent with this trend, logistic regression analyses revealed a robust inverse association between the TyG index and sarcopenia. In both unadjusted and multivariable-adjusted models, the TyG index – whether treated as a continuous, ordinal, or categorical variable – showed that for each one-quartile or one-unit increase in the TyG index, the odds of sarcopenia decreased significantly, and participants in the highest TyG quartile had approximately 30–40 % lower odds of sarcopenia compared with those in the lowest quartile.

Evidence regarding the association between the TyG index and sarcopenia remains controversial. Our results are directionally consistent with several previous studies in Chinese populations [16], [17], [18], [19], all of which reported that higher TyG levels were associated with a lower risk of sarcopenia. Notably, most prior studies were conducted in general middle-aged and older populations, whereas the present study further focused on a metabolically healthier subgroup (excluding individuals with dysglycemia and central obesity) and still observed a consistent inverse association. In contrast, studies from Korea and the United States [11], [12], [13], [14], [15] have reported a positive association, suggesting that the relationship between the TyG index and sarcopenia may be population-specific. Such discrepancies may reflect differences in underlying metabolic profiles, lifestyle factors, and genetic susceptibility across populations. Additionally, the prevalence of sarcopenia in our study (49.7 %) exceeded the 6.8–25.7 % range previously reported among older adults in Asian countries [1], 2]. Several factors may account for this discrepancy. First, our study included individuals aged 60 years and older, and the prevalence of sarcopenia increases markedly with advancing age, reaching as high as 50 % among those over 80 years [30]. Second, the relatively high proportion of rural residents in our sample may also have contributed to the higher prevalence observed, as rural residents generally have a higher risk of sarcopenia than their urban counterparts [31]. Third, ASM was estimated using an anthropometric equation rather than measured directly by DXA or bioelectrical impedance analysis (BIA), which may have introduced systematic bias. In addition, low muscle mass was defined based on the sex-specific 20th percentile rather than a fixed cutoff, potentially inflating the prevalence estimate. Notably, other studies based on the same CHARLS database have reported similar prevalence estimates (e.g., 46.0 %) [32], suggesting that our findings are comparable to those from studies using similar methods and populations.

The TyG index is calculated from fasting triglyceride and glucose. Triglycerides are the body’s major form of energy storage and also serve as an important energy substrate for skeletal muscle [20], 21]. In particular, intramuscular triglycerides provide a key fuel source during muscle contraction and exercise [21]. Notably, the role of triglycerides appears to differ substantially according to metabolic status: in metabolically healthy individuals, they function as an efficient energy source during exercise [21], whereas in the setting of insulin resistance or metabolic syndrome, excessive accumulation may promote ectopic lipid deposition within muscle (i.e., myosteatosis) [8]. In our study, the TyG index was inversely associated with sarcopenia in the metabolically healthier subgroup. One possible explanation is that, in this subgroup, triglycerides may be more likely to undergo oxidative utilization rather than ectopic storage; thus, higher triglyceride levels may reflect greater availability of energy substrates that can be mobilized by skeletal muscle to support muscle function [22], 23]. However, given the cross-sectional design, this mechanistic interpretation remains speculative and requires confirmation in longitudinal or interventional studies. In addition, reverse causation cannot be excluded. Individuals with sarcopenia may have lower triglyceride levels, and consequently a lower TyG index, because of altered body composition, insufficient dietary intake, or reduced metabolic rate. Specifically, low muscle mass may disrupt whole-body triglyceride metabolic homeostasis [33], while concomitant energy deficiency or a lower metabolic rate may further reduce fasting triglyceride levels [34], 35]. Therefore, the observed association should not be interpreted as evidence of a protective effect of the TyG index on muscle health, and future studies are needed to clarify the direction of causality.

To minimize potential confounding, we excluded participants with dysglycemia and central obesity at the study design stage, and further adjusted for waist circumference and BMI in the analyses. After these stringent controls, the TyG index remained independently and inversely associated with sarcopenia. Notably, a nationwide cohort study in China reported that the association between the TyG index and sarcopenia was no longer significant after adjustment for BMI [16]. In contrast, we still observed an independent association after BMI adjustment in the present study. We speculate that this discrepancy may be related to differences in inclusion criteria between the two study populations: by prospectively excluding individuals with central obesity and rigorously adjusting for waist circumference, our study may have more effectively controlled for obesity, particularly the confounding effect of abdominal fat accumulation, thereby allowing the potential independent association between the TyG index and sarcopenia to emerge. Additionally, a previous study of non-diabetic postmenopausal women [19] likewise reported that a higher TyG index was associated with a lower odds of sarcopenia, which is directionally consistent with our findings; however, obesity was not further excluded in that study. Taken together, these findings suggest that the inverse association between the TyG index and sarcopenia may be independent of central adiposity and dysglycemia, although this observation still requires confirmation in additional independent populations. However, several limitations should be acknowledged. Although the stringent selection criteria helped reduce confounding, they may also have introduced selection bias. The final analytic sample accounted for only 8.7 % of the original sample, potentially resulting in systematic differences from the general older population and limiting the generalizability of the findings. In addition, because the data were derived exclusively from older Chinese adults, the applicability of these findings to populations of other ethnicities or from other countries or regions remains uncertain. Future studies are warranted to validate these findings in broader and more diverse populations.

Methodologically, ASM was estimated using an anthropometric equation rather than directly measured by DXA or BIA. Although this equation was developed against DXA and validated in Chinese populations, prediction equations are inherently subject to bias and may therefore have limited generalizability. Moreover, because some input variables, such as body weight, are also associated with the TyG index and sarcopenia risk [36], 37], circularity bias cannot be excluded. Low muscle mass was also defined by sex-specific 20th percentiles within the study sample rather than standardized cutoffs, which may limit comparability across studies. In addition, nonlinear associations were not examined because of the limited sample size. Nevertheless, future studies using direct muscle measurements and larger samples are warranted. Notably, compared with previous similar studies, one strength of the present study is the use of the SPPB.

In conclusion, this study is the first to demonstrate an independent inverse association between a higher TyG index and lower odds of sarcopenia among Chinese older adults without either dysglycemia or central obesity. In this metabolically healthier subgroup, the observed association may suggest a potential link between lipid metabolism and muscle health; however, the cross-sectional design precludes causal inference. Future prospective cohort and interventional studies are needed to clarify causality and to assess the utility of the TyG index as an adjunctive marker for sarcopenia.

## Conclusions

In this CHARLS-based cross-sectional study of Chinese adults aged 60 years and older who were free of dysglycemia and central obesity, a higher TyG index was independently associated with lower odds of sarcopenia. These findings are associative rather than causal, and further prospective studies are needed to validate the TyG index’s potential utility in sarcopenia assessment.
